# Prevalence and risk factors of brain atrophy and associated confusion state among adults from three hospitals in northern Tanzania

**DOI:** 10.11604/pamj.2023.45.1.36831

**Published:** 2023-05-02

**Authors:** Leticia Joseph Kalumbilo, Emmanuel Abraham Mpolya, John-Mary Vianney

**Affiliations:** 1 Department of Health and Biomedical Sciences, School of Life Science and Bioengineering, Nelson Mandela African Institution of Science and Technology, 447 - Arusha, Tanzania

**Keywords:** Brain atrophy, confusion state, diagonal brain fraction

## Abstract

**Introduction:**

brain atrophy is the reduction of brain volume often accompanied with cognitive changes. Despite the availability of computerized-tomography (CT) scanners in Tanzania, little is known about the magnitude of brain atrophy, its associated confusion state and the risk factors in adults. This study aimed to fill those knowledge gaps.

**Methods:**

a retrospective cross-sectional hospital-based survey was conducted in northern Tanzania using a sample size of 384 CT images of adults who underwent brain CT scans in three referral hospitals. CT images were evaluated using a diagonal brain fraction (DBF) method to determine the presence of brain atrophy. Data for other covariates were also collected.

**Results:**

we report a prevalence of 60.67% for brain atrophy and 35% for the associated confusion state. Association between confusion state and brain atrophy was statistically significant (χ^2^ = 21.954, p<0.001). Brain atrophy was prognosticated by: age (adjusted OR: 1.11; 95% CI [1.05, 1.20], p<0.001), smoking (adjusted OR: 6.97; 95% CI [2.12, 26.19], p<0.001), alcohol-consumption (adjusted OR: 11.87; 95% CI [3.44, 40.81], p<0.001), hypertension (adjusted OR: 61.21; 95 CI [15.20, 349.43], p<0.001), type-2 diabetes mellitus (adjusted OR: 15.67; 95% CI [5.32, 52.77], p<0.001) and white matter demyelination (adjusted OR: 13.45; 95% CI [4.66, 44.25], p<0.001).

**Conclusion:**

there is high prevalence of brain atrophy and associated confusion state among hospitalized adults in northern Tanzania. Reported prognostic factors for brain atrophy such as age, smoking, alcohol consumption, hypertension, type-2 diabetes mellitus and white matter demyelination could help focus interventions in this area.

## Introduction

The brain is a vital organ that serves as the command center for the body's multiorgan physiological systems, receiving signals from sensory organs and sending information to other organs, such as muscles [1]. Brain atrophy (BA), also known as cerebral atrophy (CA), is a condition in which brain tissues shrink as a result of the loss of brain cells and neural connections [2,3]. Cerebral atrophy can be the result of different factors that damage neuronal and glial cells, including infection [4], head trauma [5], malnutrition [6], and incidence of ischemia [7]. Furthermore, among elderly patients with cerebral atrophy, a considerable trend of confusion state (CS) has been reported to be a prevalent outcome and described as a more serious consequence around the world [8]. A CS prevalence of nearly 20% among adults in Tanzania has been reported [9]; however, this study did not show how CS is linked to brain shrinkage. CS has been linked to an increase in morbidity among adults, as well as a burden to caregivers and society, impacting people's quality of life, causing economic disruption from the individual to the communal level [10-12].

Cerebral atrophy has become increasingly visible among Tanzanian adults due to the availability of sophisticated diagnostic imaging methods such as computerized-tomography (CT) and magnetic resonance imaging (MRI). Despite the availability of these neurodiagnostic tools, little is known about the magnitude of brain atrophy and its associated CS among adults in underdeveloped countries, including Tanzania. Furthermore, there is little evidence of complete public health awareness, mitigation plans, as well as acknowledgment of the existence of brain atrophy as an illness that continues to afflict a significant percentage of elders in society. Thus, this study intended to examine the magnitude of brain atrophy and its associated CS among adults aged fifty years and above in order to provide information about the state of brain atrophy and the associated symptoms among adults in northern Tanzania. Additionally, the study assessed the predictive factors contributing to brain atrophy in adults which provides insights into the management of senile brain atrophy. Thus, this study provides answers to the following research questions: what is the proportion of individuals with brain atrophy in adults in northern Tanzania? What is the proportion of individuals with brain atrophy presenting with confusion state? And also, what are the predictive factors of brain atrophy among adults?

## Methods

**Study design:** a cross-sectional survey through a retrospective-chart review was used to determine the prevalence of brain atrophy in association with CS, as well as the predictive risk factors for atrophy among adults in northern Tanzania.

**Setting:** this study was a hospital-based survey conducted in northern Tanzania involving three prominent hospitals with advanced imaging diagnostics, namely: the Kilimanjaro Christian Medical Centre (KCMC) in Kilimanjaro Region, the Selian Arusha Lutheran Medical Center (ALMC) Hospital and the NSK hospital, both in Arusha Region. The study involved collection of secondary data in the mentioned hospitals from March to July 2022.

**Participants:** inclusion criteria was adults aged 50 years and above who attended the radiology departments of the aforementioned hospitals for brain CT-scan from year 2019 to 2021. Exclusion criteria were adults with space-occupying lesions, tumors and any deformation in their brain anatomy.

**Variables:** both quantitative and qualitative variables were collected in this study. Quantitative variables involved age and measurement of diagonal brain fraction formula (DBF) value to determine the presence or absence of brain atrophy. Qualitative variables collected were those determining the presence of CS, binary variables such as alcohol-consumption, smoking, traumatic brain injury (TBI), hypertension, type-2 diabetes mellitus (T2DM), CT-evidence of white matter demyelination, and CT-evidence of paranasal sinus infection to identify the risk factors of an outcome variable which was brain atrophy. Brain atrophy was further classified based on severity into four levels: normal, mild, moderate and severe. Details of how the four levels were determined is provided in the next section.

**Figure 1:**
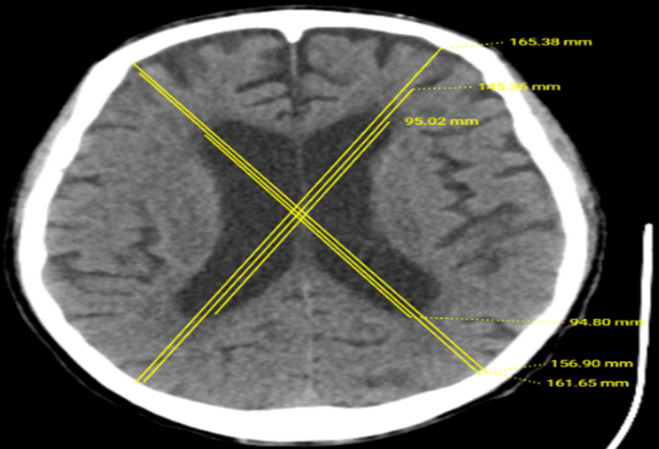
brain axial view at the level of the widest part of the lateral ventricle; CT-scan image indicating measurement as per DBF standards

**Data sources/measurement of variables:** the study involved the collection of brain CT-scan images, which were already scanned using 6, 16 and 128 slices using Siemens Somatom Emotion, Sensation and Definition machines respectively, with a slice thickness of 5mm and an increment of 2mm. From Figure 1, a computer-assisted system was used to measure the brain volume of all the images using a DBF formula designed to measure the dimensions of the brain relative to the skull and ventricles [13]. This study considered cut-off for a DBF value of 0.75 to describe the presence of brain atrophy. Thus, a value <0.75 indicated the presence of atrophy, and a value >0.75 indicated normal brain volume. Additionally, to grade the severity of brain atrophy, this study considered the range and grade of brain atrophy as follows: 0.15 - 0.30 (very severe), 0.31 - 0.45 (severe), 0.46 - 0.60 (moderate), 0.61 - 0.75 (mild), and >0.75 (normal brain volume) [13].

The presence of a CS was confirmed by patients having these signs in their medical history: disorientation to time, place, and persons; refusal to speech; aggressiveness, and memory loss. Additionally, to determine the drivers of brain atrophy, secondary data were collected through review of the medical history of a patient. This involved collection of variables such as age, TBI, alcohol-consumption, smoking, hypertension, T2DM, CT-evidence of white matter demyelination, and CT-evidence of paranasal infection.

**Bias:** this study is an observational retrospective hospital-based survey in which the control of selection bias involved simple-random sampling of hospital records. Random selection of already scanned CT-images of patients along with files containing their medical history was done and every response in this study was acquired from patient's file. There were no missing values which eliminated bias in the outcome measures. No questions of past exposures were asked to participants directly which eliminated any recall bias. Moreover, as no interviews were done, there was no interviewer or social acceptability biases.

**Study size:** the sample size was calculated using the Kish and Leslie formula; n = Z^2^pq/d^2^ [14], in which the standard normal deviate Z, corresponding to the 95% confidence interval was 1.96, due to absence of evidence of the proportion of brain atrophy in Tanzania a 50% prevalence, p, of brain atrophy in adults aged 50 years and above was used, which has the maximum variance in a binomial distribution, this proportion gives the maximum sample size. Finally, the degree of precision, d, was taken as 5%, our estimates would be within 5% of the true population parameters. The sample size was 384 adults who underwent a brain CT-scan. From a rule of thumb that at least one variable in the regression model must have at least 10 hospital charts, this sample size provided about 38 hospital charts per variable, ensuring enough power of our regression and multinomial models which only had 9 variables.

**Quantitative variables:** there were only two quantitative variables: age and DBF value. Age was collected from the hospital charts while DBF was a derived variable calculated according to Sungura, *et al*. 2020 [13]. As already described in the data source section, this study considered the cut-off for a DBF value of 0.75 to describe the presence or absence of brain atrophy. Additionally, to grade the severity of brain atrophy, this study considered the range and grade of brain atrophy as follows: 0.15 - 0.30 (very severe), 0.31 - 0. 45 (severe), 0.46 - 0.60 = (moderate), 0.61 - 0.75 (mild), and >0. 75 (normal brain volume). In our data there were no participants with very severe brain atrophy and so only four levels are analyzed.

**Statistical methods:** data were analyzed by the R-statistical tool [15]. Categorical data obtained were summarized using counts and percentages while continuous variables were summarized using mean, median and quantiles. Secondly, a cross-tabulation between brain atrophy and various categorical variables was done with the calculation of the chi-squared statistics to establish if there were any dependence between brain atrophy and a particular variable. Thirdly, univariable logistic regression (for obtaining crude odds ratios) and multivariable logistic regression (for obtaining adjusted odds ratios) were done to establish prognostic factors for brain atrophy. All variables were included in both the univariable and the multivariable logistic regressions. Results are summarized as crude and adjusted odds ratios (ORs) respectively with their p-value and 95% confidence intervals. A p-value of 5% was used as a cut-off for statistical significance.

Finally, a multinomial regression was done to establish the role of various factors in determining the severity of brain atrophy, results are reported in terms of the coefficients of different predictive factors with their 95% confidence intervals and p-values. No subgroup or interaction analyses were conducted as we assumed that the standard of care was the same for all individuals in the three hospitals. This study had no missing data and sensitivity analysis was not involved as this study followed only one group of adult population.

**Ethics statement:** ethical clearance for this study was obtained from the KNCHREC with reference number: KNCHREC00063/01/2022). In extracting patients' information from the hospital files, data was collected without taking the patient's names to ensure their privacy. An anonymous identifier was used. There was no direct interaction with patients therefore no harm or informed consent during data collection. However, permission to access and extract patient's information was duly sought and granted by the three study hospitals.

## Results

**Participants:** the total population in this study included 384 patients who visited the radiology departments of the referral health facilities in northern Tanzania. It being a hospital charts review there was no missing number of participants.

**Descriptive data:** results in Table 1 shows that mean age of the study population was 70 years and the population was divided into five age groups: 50-60, 61-70, 71-80, 81-90 and more than 90 years. The predominant population involved adults with an age group of 71-80 years old, while the group of 90 years and above constituted a relatively lower population. Gender distribution of the population revealed a total of 205 males and 179 females, representing 53.4% and 46.6% of the patients respectively. Also, results showed that KCMC had 159 (41.4%) patients, NSK had 155 (40.4%) patients and Selian Hospital had 70 (18.2%) patients. Therefore, KCMC had many patients compared to the other two hospitals as it is a zonal referral hospital in the northern zone.

**Table 1:** demographics, severity of atrophy, CSs and other predictors

Variable	Levels	n(%)	Mean	Median	Q25%	Q75%
Age			70.3	70	60	79
Age groups	50-60	91 (24)				
	61-70	96 (25)				
	71-80	104 (27)				
	81-90	68 (18)				
	Above 90	25 (7)				
Gender	Female	179 (47)				
	Male	205 (53)				
Health facility	ALMC	70 (18.2)				
	KCMC	159 (41.4)				
	NSK	155 (40.4)				
**DBF Values**			0.698	0.71	0.62	0.78
State of atrophy	Atrophied	232 (60)				
	Normal	152 (40)				
Severity of brain atrophy	Normal	161 (42)				
	Mild	139 (36)				
	Moderate	76 (20)				
	Severe	8 (2)				
Confusion state	Yes	128 (33)				
	No	256 (67)				
Traumatic brain injury/head trauma	Yes	138 (36)				
	No	246 (64)				
Smoking	Yes	171 (45)				
	No	213 (55)				
Alcohol-consumption	Yes	284 (74)				
	No	100 (26)				
Hypertension	Yes	275 (72)				
	No	109 (28)				
Type 2 Diabetes	Yes	220 (57)				
	No	164 (43)				
White matter-demyelination	Yes	224 (58)				
	No	160 (42)				
Paranasal infection	Yes	169 (44)				
	No	215 (56)				

### Outcome data and main results

**Figure 2:**
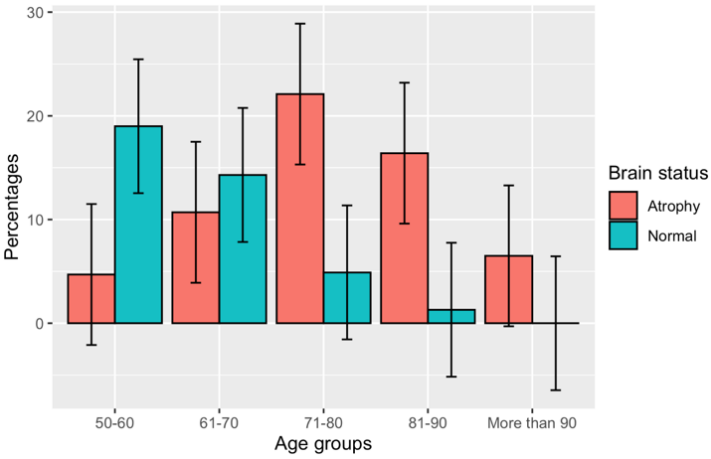
percentage of brain atrophy among study participants against age categories as grouped in the population included in this study

**Figure 3:**
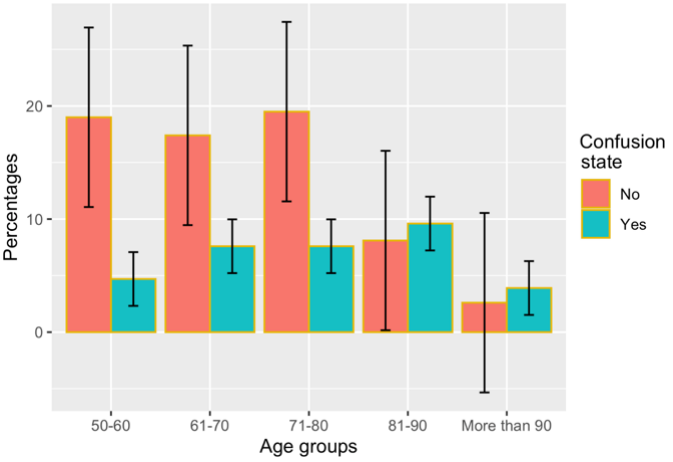
percentage of confusion state among study participants against age categories as grouped in the population included in this study

**Prevalence of brain atrophy, state of severity and confusion state:** the outcome data in this study was the state of brain atrophy which was coded as a binary variable (present = 1, absent = 0). Results in Table 1 shows that in this study; 232 (60%) adults presented with brain atrophy, and the remaining 152 (40%) adults showed normal brain volume or had other brain pathologies not related to brain atrophy. The mean DBF value was 0.698, its median being 0.71 (IQR: 0.62 - 0.78). Also, our findings revealed a severity grade of brain atrophy from mild to severe. The grade of brain atrophy appeared to be most prevalent was in the mild-grade with a prevalence of 36%, followed by the moderate-grade 20% and the severe-grade 2%. Moreover, Figure 2 indicated brain atrophy occurs across all age-categories in the study population. However, brain volume loss increased as the age of the participants increased. Additionally, our results revealed that from the total population, 128 (33%) patients presented with a confused state (altered mental status). Figure 3 indicated CS to occur across all age-categories in the study population, and the percentage of CS compared to non-confused increased as the age of the participants increased.

**Association between brain atrophy and confusion state as well as predictive factors:** Table 2 summarises results of cross-tabulation between brain atrophy and several categorical variables and the calculation of the test for dependency using the chi-squared statistics. Across all categorical variables involved it reveals that they have a statistically significant dependence with brain atrophy (p<0.05). Thus, in the population of the patient diagnosed with brain atrophy, the condition of being confused is significantly related with brain atrophy. Also, our results showed that out of 205 male patients who were included in this study and whose brain CT-scan images were reviewed, 136 (35%) patients had reduced brain volume, and of 179 female patients who were included in this study, 96 (25%) patients had reduced brain volume. Thus, brain atrophy was dependent on the gender (p<0.05). In fact, Table 2 shows that all other predictive factors including age, TBI, smoking, alcohol-consumption, hypertension, T2DM, white matter demyelination and paranasal sinus infection, showed a statistically significant dependence with brain atrophy (p<0.001).

**Table 2:** association between predictive factors and brain atrophy

Variable		Atrophy: n(%)		Chi-squared value	p-value
		Yes	No		
Confusion state	Yes	99 (26%)	29 (8%)	21.954	<0.001
	No	133 (35%)	123 (32%)		
Gender	Female	96 (25%)	83 (22%)	5.9347	<0.05
	Male	136 (35%)	69 (18%)		
Age-groups	50-60	18 (5%)	73 (19%)	141.1	<0.001
	61-70	41 (11%)	55 (14%)		
	71-80	85 (22%)	19 (5%)		
	81-90	63 (16%)	5 (1%)		
	Above 90	25 (7%)	0 (0%)		
Head trauma	Yes	101 (26%)	37 (10%)	13.871	<0.001
	No	131 (34%)	115 (30%)		
Smoking	Yes	136 (35%)	35 (9%)	45.673	<0.001
	No	96 (25%)	117 (30%)		
Alcohol-consumption	Yes	200 (52%)	84 (22%)	44.063	<0.001
	No	32 (8%)	68 (18%)		
Hypertension	Yes	229 (60%)	46 (12%)	208.27	<0.001
	No	3 (1%)	106 (28%)		
Type-2 Diabetes	Yes	194 (51%)	26 (7%)	163.34	<0.001
	No	38 (10%)	126 (33%)		
White matter demyelination	Yes	191 (50%)	33 (9%)	136.35	<0.001
	No	41 (11%)	119 (31%)		
Paranasal infection	Yes	75 (20%)	94 (24%)	31.278	<0.001
	No	157 (41%)	58 (15%)		

percentages are rounded to 0 decimal places

**Influence of predictive factors for brain atrophy among adults in northern Tanzania:** Table 3 summarises results from univariable and multivariable logistic regressions to determine the influence of different variables on brain atrophy. The outcome variable was the state of brain atrophy (present = 1, absent = 0). On a univariable analysis, results revealed that nearly all the predictive factors studied in this investigation had a significant impact on influencing the prognosis of brain atrophy among adults in northern Tanzania with crude odds ratio (cOR) >1; p<0.05, which means the odds of brain atrophy were higher with the particular predictor. However, an exception was the paranasal infection whose odds ratio was not statistically significant (cOR = 0.28; 95% CI = [0.91,10.54]; p>0.05). Results of the multivariable logistic regression provided us with adjusted odds ratios. In a multivariable framework age showed to be a significant risk factor for brain atrophy among the study patients increasing the odds of brain atrophy by 11% for each unit increase in age (aOR =1.11; 95% CI = [0.91,10.54]; p<0.001). In a multivariable model, traumatic brain injury was not a statistically significant predictor of brain (aOR =2.84; 95% CI = [0.797,10.73]; p>0.05). Smoking is a strong prognostic factor for brain atrophy increasing the odds of a brain atrophy almost seven times among those who smoke compared to non-smokers (aOR =6.97; 95% CI = [2.12,26.19]; p<0.01). Furthermore, adults who were previously exposed to alcohol-consumption were 11.87 times more likely to have brain atrophy than those who were not exposed to alcohol (aOR =11.87; 95% CI = [3.44,40.81]; p<0.01). Adults with hypertension are 61.21 more likely to have brain atrophy than those who are not hypertensive (aOR =61.21; 95% CI = [15.20,349.43]; p<0.001). Judging from the 95% Cis, the risk for brain atrophy associated with hypertension can range between 15 times to up to 350 times compared to those without hypertension. With an aOR of 15.67, adults who had T2DM were 15.67 times more likely to have brain atrophy than those who did not have T2DM (aOR =15.67; 95% CI = [5.32,52.77]; p<0.001). Adults who already developed white matter lesions were 13.45 times more likely to have brain atrophy than those who did not have white matter demyelination (aOR = 13.45; 95% CI = [4.66,44.25]; p<0.001). However, the relationship between paranasal sinus infection and brain atrophy among adults in northern Tanzania was not statistically significant (aOR = 2.90; 95% CI = [0.91,10.54], p>0.05).

**Table 3:** univariate and multivariate logistics regression model for risk factors influencing brain atrophy (1 = atrophy, 0 = normal)

Variable	Levels	cOR	95% CI [LL, UL]	p-value	aOR	95% CI [LL, UL]	p-value
Age		1.15	[1.12, 1.19]	<0.001	1.11	[1.05,1.20]	<0.001
Traumatic Brain Injury	No	Ref					
	Yes	2.35	[1.50, 3.73]	<0.001	2.84	[0.797,10.73]	>0.05
Smoking	No	Ref					
	Yes	4.91	[3.12, 7.88]	<0.001	6.97	[2.12, 26.19]	<0.01
Alcohol-consumption	No	Ref					
	Yes	4.83	[2.99, 7.95]	<0.001	11.87	[3.44, 40.81]	<0.001
Hypertension	No	Ref					
	Yes	180.59	[64.31, 756.44]	<0.001	61.21	[15.20, 349.43]	<0.001
Type 2 diabetes	No	Ref					
	Yes	25.86	[15.14, 45.79]	<0.001	15.67	[5.32, 52.77]	<0.001
White matter demyelination	No	Ref					
	Yes	16.26	[9.88, 27.47]	<0.001	13.45	[4.66, 44.25]	<0.001
Paranasal infection	No	Ref					
	Yes	0.28	[0.19, 0.44]	<0.001	2.90	[0.91, 10.54]	>0.05

cOR = crude odds ratio, aOR = adjusted odds ratio, CI = confidence interval, LL = Lower level, UL = Upper level

**Predictors for severity state of brain atrophy:** Table 4 summarises the multinomial regression results to assess the influence of study variables on the severity of brain atrophy. Only four levels of severity were available in our study: normal, mild, moderate and severe. These four levels were the outcomes of our multinomial regression models. All predictor variables included in the logistic regression models in the section above were also included in the multinomial regression models. Coefficients of explanatory variables were extracted as well as their 95% confidence intervals and p-values. The null value of the coefficient is the zero value and so confidence intervals including this null value will not be statistically significant. For each coefficient, its effect is additive for a unit change in the explanatory variable. Table 4 shows that age has a positive effect in affecting all severity levels above normal (p < 0.001). Confusion state and head trauma are not predictors of any level of severity of brain atrophy (p > 0.05). Smoking has effects on determining mild and moderate brain atrophy (p < 0.05) and has no effect in determining severe brain atrophy (p > 0.05). On the other hand, alcohol consumption seems to have an effect in positively determining all levels of severity (p < 0.05). With similar effect of affecting all levels of severity are hypertension (p < 0.05), type 2 diabetes mellitus (p < 0.05) and white matter demyelination (p < 0.05). Table 4 details the actual coefficients and their 95% confidence intervals.

**Table 4:** multinomial regression results for predictors of severity of brain atrophy

Predictor variable	Outcome variables (Levels of severity of brain atrophy)	Coefficient	95% CI [LL, UL]	p-value
**Age**	**Normal**	**Ref**		
	Mild	0.085	[0.038, 0.133]	<0.001
	Moderate	0.153	[0.097, 0.208]	<0.001
	Severe	0.254	[0.142, 0.367]	<0.001
**Confusion (Yes)**	**Normal**	**Ref**		
	Mild	-0.422	[-1.435, 0.591]	>0.05
	Moderate	-0.391	[-1.508, 0.726]	>0.05
	Severe	1.495	[-0.943, 3.933]	>0.05
**Head Trauma (Yes)**	**Normal**	**Ref**		
	Mild	0.515	[-0.474, 1.504]	>0.05
	Moderate	0.530	[-0.576, 1.636]	>0.05
	Severe	0.984	[-0.904, 2.872]	>0.05
**Smoking (Yes)**	**Normal**	**Ref**		
	Mild	1.079	[0.156, 2.001]	0.022
	Moderate	1.536	[0.482, 2.590]	0.0043
	Severe	1.203	[-0.715, 3.121]	>0.05
**Alcohol consumption (Yes)**	**Normal**	**Ref**		
	Mild	1.786	[0.827, 2.745]	0.000263
	Moderate	1.253	[0.137, 2.369]	0.028
	Severe	24.253	[21.942, 26.564]	<0.0001
**Hypertension (Yes)**	**Normal**	**Ref**		
	Mild	3.36	[1.98, 4.75]	<0.0001
	Moderate	25.27	[23.08, 27.46]	<0.0001
	Severe	10.00	[7.69, 12.31]	<0.0001
**Type 2 Diabetes Mellitus**	**Normal**	**Ref**		
	Mild	1.57	[0.67, 2.47]	<0.001
	Moderate	1.82	[0.69, 2.96]	0.002
	Severe	25.20	[22.89, 27.51]	<0.0001
**White matter demyelination**	**Normal**	**Ref**		
	Mild	2.08	[1.21, 2.94]	<0.0001
	Moderate	2.29	[1.20, 3.39]	<0.0001
	Severe	1.17	[-1.46, 3.79]	0.03
**Paranasal infection**	**Normal**	**Ref**		
	Mild	0.96	[-0.01, 1.93]	>0.05
	Moderate	0.93	[-0.18, 2.03]	>0.05
	Severe	0.09	[-2.38, 2.56]	>0.05

## Discussion

### Key results

**Prevalence of brain atrophy and the associated confusion state:** in this study, we used a hospital-based sample of adults aged 50 years and above to report the prevalence of brain atrophy among adults in northern Tanzania. To our knowledge, this is the first study to establish the prevalence of brain atrophy conditions among adults in Tanzania. The study reports a high prevalence of brain atrophy from the study population in northern Tanzania. Male population constitutes higher proportion of brain atrophy than female: thus, the study showed a sex difference in the changes in brain volume. This higher prevalence of brain atrophy can be anticipated and may be accelerated by pathologies such as stroke [16], alcoholic encephalopathy [17], liver cirrhosis [18], diabetes [19], and as reported by other studies of brain atrophy cases. Additionally, our results are consistent with other CT-scan-based published studies that report that there is more brain atrophy with aging in men than in women [20-24].

In this study, brain atrophy was more prevalent in adults aged 60 years and above, and the likelihood of finding adults with normal brain volume decreased as the age of the individual increased. Therefore, the adults were concluded to develop brain atrophy conditions that are highly associated with age. These findings are consistent with the discovery that the incidence of brain atrophy in association with neuro-diseases such as Alzheimer's Disease (AD) increases exponentially with age after the age of 65 [25,26], indicating that age has the greatest effect on the development of cerebral atrophy in adults [27]. In the elderly, brain shrinkage is strongly linked to local cellular composition as well as the gradual aggregation of neurotoxic proteins and waste products that do not drain into the glymphatic system [27]. The combination of metabolic slowing and decreased cellular regeneration causes structural and functional degeneration to the brain, which contributes to cognitive decline [28].

Furthermore, the state of confusion among adults was related to brain atrophy. CS appeared to dominate with equal frequency across both male and female, with the tendency of increasing frequency as age increased. Our results constituents by other reports, which show the influence of cerebral atrophy on confusion among adults regardless of the sex of the population [29]. Additionally, not every case of confusion was associated with brain atrophy, and patients with normal brain volume also show an incidence of confusion. Thus, to the group of populations consisting of patients with normal brain volume, CS might be related to other factors that are not associated with brain atrophy, such as hypertensive encephalopathy [30], TBI [31], infection [32], and adverse drug effects [33]. Since the rate of total brain atrophy varies among individuals and importantly associated with age-related losses in cognitive function, the overall burden of brain atrophy among hospitals and societies in Africa increases. Brain atrophy in association with CS stand as the leading cause of nursing home placement and a major health economic burden to family members and caregivers [29].

**Influence of predictive factors for brain atrophy:** after adjustment for all predictive factors of brain atrophy in our study, hypertension was a powerful determinant for cerebral atrophy than all other predictive factors. Reports show a steep and continuous linear relationship between high blood pressure and the risk of stroke [34]. Individuals with hypertension can develop different types of strokes, including ischemic, intracerebral and subarachnoid haemorrhages, which damage the brain cell [35]. A long-standing hypertension results in structural changes in the brain which results to brain cell death and dementia [35]. Thus, hypertension not only worsens the initial injury due to stroke but also exacerbates ongoing tissue damage to the brain tissue. With the association between T2DM and cerebral atrophy, our results show patients with diabetes appear to be at high risk of developing brain atrophy. Our results are consistent with other research that reports T2DM to be associated with an increased risk of incident of dementia and Alzheimer's disease, which eventually develop brain atrophy [36]. Other studies describe mixed results, some reporting T2DM to be associated with greater rates of increase in ventricular volume but not in decline of total brain volume over time [37]. Additionally, evidence shows T2DM to closely be related with cerebral small-vessel diseases, which are a key factor for the induction of silent brain injury that appears around the cerebral ventricles called white matter hypo intensities (WMHs) at older ages [38].

In this study, WMHs show a significant association with cerebral atrophy, and the significant influence of T2DM in this study can be explained by the high rate of patients having hypertension, T2DM, and WMHs in concurrent manner. Our review also reveals alcohol to play part in influencing the incidence of brain atrophy, patients with history of alcohol-consumption are at high risk of developing brain atrophy. This is supported by researchers who report alcoholic brain atrophy to be associated with reduced cerebral blood flow and glucose metabolism [39], which eventually damage some parts of the brain [40]. Also, morphometric analysis of post-mortem brains from chronic alcohol users shows continuous loss of brain cells and regional distribution atrophy in the cerebral hemispheres of alcoholics [41]. Therefore, an association between alcohol-consumption and brain atrophy is evident at the population level.

Reports show that, whole-brain atrophy occurs after mild or moderate TBI and is evident at an average of 11 months after trauma [42]. Our report shows that TBI contributes to incidence of brain atrophy later in life. Although previous volumetric imaging studies have demonstrated the presence of brain atrophy after TBI [43], TBI is associated with an increased risk of neurodegenerative disease, including AD, Parkinson's disease and chronic traumatic encephalopathy, leading to brain atrophy [44]. In adult population, this leads to cognitive impairments such as loss of memory, and executive dysfunction, which in turn increase dependence [45].

Furthermore, we report adults population who are exposed to smoking are at high risk of having brain atrophy. Reports have shown that alcohol use disorders show an intricate interplay between smoking and alcohol use, which eventually leads to damaged brain tissues [46]. However, findings on which brain structures may preferentially be affected by smoking are not well explored in this study due to methodological limitations; generally, this can be explained by reports showing smokers to exhibit structural changes in some brain areas in comparison to non-smokers [47].

In contrast, we report adult population in northern Tanzania to have brain atrophy which is not associated with paranasal infection. Although, the enlargement of the paranasal sinuses, mastoid cells and calvarial thickening have been reported in patients with cerebral hemi-atrophy [48,49]. As the pathogenesis of sinusitis to the central nervous system (CNS) has not been well explored globally. We suggest further studies regarding types of paranasal infection and their effect on the CNS be carried out and study the mechanism by which these infections affect brain cells.

Finally, in establishing grades of brain atrophy, the condition is more dominant at the mild stage, followed by the moderate stage and then the severe stage. Thus, the brain atrophy among adult population in northern Tanzania is still at an early stage (mild stage). Additionally, this study provides a mark showing age of individual to influence at most the severe-grade of brain atrophy among patients. Most importantly, our results reveal CS and head trauma to not have any influence on severe-grade of brain atrophy, thus, we cannot consider CS and head trauma as prognostic factors to be used as justification for severity of brain atrophy during brain atrophy diagnosis in the hospitals. On the other hand, smoking, alcohol-consumption, hypertension, T2DM and white matter demyelination showed to have a significant impact on all levels of severity grade of brain atrophy as the model coefficients increased from mild-grade to severe-grade. This show that the longer the lasting of the mentioned risk factors the more severe the condition of brain atrophy becomes. Paranasal infection showed to only have impact at mild and moderate-grade but cannot be used as a significant influential factor to cause the severe-grade of brain atrophy.

**Limitations:** given that brain atrophy appears to be related to cognitive ability and increased burden of dependence in the elderly to the caregivers and society, a reasonable course for future studies need to involve further exploration of brain atrophy management through social and clinical interventions. We only considered individuals in late adulthood age strata. Future studies should address the magnitude of atrophy and the impact of the associated CS on cognition across a more diverse age range, including middle-aged (30 - 40 years) individuals.

**Generalisability:** this study involved a sample population of adults aged 50 years and above who were performed a brain CT-scan in three major hospitals in northern Tanzania. Our methodology, which is retrospective medical records review can be replicated elsewhere and the reported results are generalizable to that particular population elsewhere.

## Conclusion

We report a high prevalence of brain atrophy among aged adult while confusion state appears to be a major associated symptom of the condition. We confirm age to be the major factor among brain atrophy cases found in northern Tanzania. Other prognostic factors are hypertension, type 2 diabetes mellitus, white matter demyelination, smoking and alcohol-consumption. These prognostic factors are also responsible for differential determination of the severity of brain atrophy. These insights about the role of prognostic factors should form the ground for improved interventions to minimize the possibilities of brain atrophy and its associated confusion states. We further recommend that future studies should focus on exploring brain atrophy with regards to specific regions of the brain and studying these region-specific atrophy patterns in response to the identified risk factors.

### What is known about this topic

- *Brain atrophy is a condition affecting the elderly population, especially at the age of 50 years and above*.

### What this study adds

- *Quantifies for the first time the prevalence of brain atrophy in Tanzania among the adult population as being 60.67%;*
- *Reports on other prognostic factors in addition to age as: hypertension, type 2 diabetes mellitus, white matter demyelination, smoking and alcohol-consumption;*
- *Reports the differential roles of such prognostic factors in determining the severity of brain atrophy*.
